# Tumor-Specific T Cell Activation in Malignant Brain Tumors

**DOI:** 10.3389/fimmu.2020.00205

**Published:** 2020-02-13

**Authors:** Malte Mohme, Marian Christoph Neidert

**Affiliations:** ^1^Department of Neurosurgery, University Medical Center Hamburg-Eppendorf, Hamburg, Germany; ^2^Department of Neurosurgery, Clinical Neuroscience Center, University Hospital and University of Zurich, Zurich, Switzerland; ^3^Department of Pathology and Center for Cancer Research, Massachusetts General Hospital and Harvard Medical School, Boston, MA, United States; ^4^Broad Institute of Harvard and MIT, Cambridge, MA, United States

**Keywords:** immunity, T cells, glioblastoma, glioma, vaccine, tumor-specific, immunotherapy, tumor-infiltrating lymphocytes

## Abstract

Due to their delicate locations as well as aggressive and infiltrative behavior, malignant brain tumors remain a therapeutic challenge. Harnessing the efficacy and specificity of the T-cell response to counteract malignant brain tumor progression and recurrence, represents an attractive treatment option. With the tremendous advances in the current era of immunotherapy, ongoing studies aim to determine the best treatment strategies for mounting a tumor-specific immune response against malignant brain tumors. However, immunosuppression in the local tumor environment, molecular and cellular heterogeneity as well as a lack of suitable targets for tumor-specific vaccination impede the successful implementation of immunotherapeutic treatment strategies in neuro-oncology. In this review, we therefore discuss the role of T cell exhaustion, the genetic and antigenic landscape, potential pitfalls and ongoing efforts to overcome the individual challenges in order to elicit a tumor-specific T cell response.

## T Cell Activation and Exhaustion

### Basics of T Cell Activation and Antigen Recognition

Eliciting a tumor-specific T cell response is the primary goal of most immunotherapeutic treatment strategies. Two major CD3^+^ T cell populations exist—CD4^+^ T Helper cells and CD8^+^ cytotoxic T cells (CTLs). While CD4^+^ T cells are able to orchestrate and modulate an antigen specific immune response through their high plasticity and ability to produce cytokines, CTLs can induce selective cell apoptosis through direct cell-cell interaction and targeted release of effector molecules, such as perforin and granzymes. The specificity of T cells is guided through activation of the T cell receptor (TCR) which recognizes antigens in the form of peptides, which are presented by human leukocyte antigen (HLA) molecules on the cell surface (1). Although reasonable cross-presentation of antigens, as well as cross-recognition by TCRs has been described in all T cell populations, CTLs primarily interact with peptide/HLA class I molecule complexes, which are expressed by all nucleated cells and present intracellular antigens. CD4^+^ T cells on the other hand are mainly activated by antigens presented by HLA class II molecule peptide complexes, which are expressed on antigen presenting cell populations, i.e., dendritic cells, macrophages/microglia and B cells (2). HLA class II molecules present mainly extracellular antigens. In general, antigens are presented in form of 8–10 (HLA class I) and 12–15 (HLA class II) amino acid long peptides, respectively, after their protein of origin has been degraded by the proteasome in the cytosol and loaded onto HLA molecules in the endoplasmic reticulum (3).

Unfortunately, since tumor cells are derived from normal tissue, antigenic demarcation represents a major hurdle for tumor-specific immune initiation (2). Here, tumor-specific antigens (TSA), describing antigens which are found exclusively in tumor cells, i.e., due to tumor-specific gene expression, or tumor-associated antigens (TAA), which are not exclusive, but may be aberrantly expressed in tumor cells, represent the desired target to stimulate a tumor-directed immune response. Upon recognition of the tumor-antigen through TCR/peptide:HLA ligation, T cell activation requires additional stimuli from co-receptors (1). These co-receptors can promote or inhibit T cell activation, and tumors might benefit from expressing inhibitory receptors, which are also known as checkpoint molecules. The interplay of antigen presentation and co-receptor expression highlights the functional complexity of the immunological synapse during tumor-specific T cell responses. In this review, we will therefore summarize the current state of the literature and discuss potential treatment strategies to elicit a tumor-specific T cell response in malignant brain tumors.

### Tumor-Infiltrating Lymphocytes in Malignant Brain Tumors

Tumor-infiltrating lymphocytes (TIL) are primarily composed of CD8^+^ CTLs, conventional CD4^+^ T helper cells and regulatory T cells (T_regs_), which in most studies are defined by CD4^+^ CD25^+^ FoxP3^+^ expression (4–6). Although CD3^+^ T cell infiltration is vastly outnumbered by tumor-associated macrophages and microglia, multiple studies have proven that increased tumor-infiltration with T cells is associated with prolonged survival in glioblastoma (4, 7–9). T cell-associated survival in glioblastoma patients was independent of age, post-operative treatment, and MGMT promotor methylation status (7). However, although a positive correlation of TILs with overall survival was shown in multiple cancer entities, this observation is still discussed controversially in glioblastoma. For example, a study by Zhai et al. demonstrated that higher CD3e/CD8a mRNA expression levels correlated with decreased survival in glioblastoma (10). In addition, the role of different T-cell subsets in the orchestration a tumor-specific immune response is still incompletely understood. Here, the study by Ladomerski et al. showed that depletion of CD8^+^ T cells at a late time-point during tumorigenesis and treatment in a syngeneic murine glioma model leads to a loss of treatment effect, while CD4+ T cells were indispensable at every time-point (11). Notably, this effect implicates an early tumor-infiltration of CD8^+^ T cells and a continuous impact of CD4^+^ T cells throughout the disease course. Furthermore, a recent comprehensive analysis of published gene expression data by us showed that individual mRNA gene expression levels need to be interpreted cautiously as immune cell subsets should better be evaluated using a gene expression signature (12). Here, our study by Bockmayr et al. demonstrated a positive correlation of infiltrating T cells, defined by a gene signature consisting of 10 mRNA expression levels, with overall survival in *IDH*-wildtype glioblastoma (12).

The prevalence of T cell subsets increases with tumor grade in astrocytic tumors (4). Interestingly, this finding was not confirmed in high-grade meningioma, as the number of infiltrating T cells decreased in anaplastic meningioma (WHO °III) compared to WHO grade I tumors (13). Furthermore, correlative analysis of TIL numbers in pediatric medulloblastoma showed no association of an increased lymphocyte infiltration with prolonged survival (14). However, difference in immune infiltration could be observed between different medulloblastoma subtypes, with SHH tumors having the strongest infiltration of T cells (15). The study even described that patients with higher numbers of granzyme B-positive CTLs, i.e., activated CD8^+^ cells, had a shorter survival compared to patients with low granzyme B-positive CTLs (14). Comparison of the immunophenotype of different pediatric brain tumors demonstrated significantly higher infiltration of myeloid and lymphoid cells in pilocytic astrocytoma and ependymoma compared to malignant tumors, such as medulloblastoma or glioblastoma (16). It is not clear if the disparity between grade-dependent increase of immune infiltrates in astrocytic tumors compared to low TIL numbers in meningioma or malignant pediatric tumors can be explained, for example, due to the underlying differences in genetic aberrations or due to differences in the mechanisms of immune escape.

### Tregs—Opposing Tumor-Specific Immune Activation

Within the tumor-specific TIL population, regulatory T cells (T_regs_) were identified as a pro-tumorigenic subpopulation. Characterized by their hallmark cytokines, IL-10 and TGF-β, which can effectively suppress tumor-specific T cell activation, T_regs_ are attracted to the local tumor environment by soluble mediators, such as CCL22 or CCL2 produced by glioblastoma cells (17–19). Higher tumor-grade was paralleled by increasing T_reg_ infiltration in glioma (20). Although discussed controversially, increased T_reg_ frequencies correlate with shorter survival and earlier recurrence in glioblastoma patients (9, 21). This finding, however, could not be confirmed when screening for mRNA expression of FoxP3 and Helios, both T_reg_-specific transcription factors, in large glioblastoma TCGA data sets (22). Recently a similarly immunosuppressive CD4^+^ FoxP3^−^ subpopulation was identified, called Type 1 regulatory T cells (Tr1) (23). Tr1 cells co-express the surface markers CD49b and Lag-3 and are upregulated in peripheral blood and in the tumor microenvironment in glioblastoma patients (24). In medulloblastoma and other malignant brain tumors, studies on the involvement of T_regs_ in tumor progression are scarce. Gene expression studies found a higher T_reg_ marker expression in the WNT medulloblastoma subtype (15). A recent investigation in medulloblastoma tissue, showed that a tumor driving pathway, e.g., mTOR activation can cross-talk with indolamin-desoxygenase (IDO) expression, which consecutively induces T_reg_ cell expansion and immunosuppression of tumor-specific immune responses (25). The IDO-mediated immunosuppression through recruitment of T_regs_ with a subsequent negative impact on overall survival was confirmed in glioblastoma patients (26). Therefore, the direct interaction between tumor-specific CTLs and T_regs_ will represent a major focus for immune monitoring protocols in current and upcoming immunotherapeutic strategies.

### T Cell Exhaustion in Glioblastoma

During tumor evolution, cancer cells develop strategies to escape from tumor-specific immune elimination (27). As the best studied example in malignant brain tumors, we will discuss T cell exhaustion in the context of glioblastoma. As mentioned above, glioblastoma attract immunosuppressive immune cell populations, such as MDSC or T_regs_, which secrete soluble mediators to silence the tumor-directed immune response. However, cancer cells themselves can express negative regulators of immune activation. Together, multiple mechanisms lead to the inhibition of T cell activation, consequently resulting in dysfunctional and exhausted T cells. The most prominent example of direct TIL inhibition in glioblastoma is the immune checkpoint programmed cell death ligand 1 (PD-L1). PD-L1 expression was observed in 2.8–32.2% of glioblastoma tumors, with a high variability due to differences in quantification and antibody usage (28–30). The relevant PD-L1 expression and negative correlation with overall survival paved to way for the first immune checkpoint studies in glioblastoma (29–31). In parallel, studies increasingly focused on characterizing the effect of immune escape on T cells. T cell exhaustion in glioblastoma at first described dysfunctional immune phenotypes in peripheral blood. Here, gene expression profiling of peripheral T cells from glioblastoma patients revealed down regulation of genes involved in TCR ligation, activation and intracellular signaling compared to healthy controls (5). Furthermore, the frequency of CD4^+^ CD57^+^ T cells and negativity for the activation marker CD28 could be correlated with survival in HCMV positive glioblastoma patients (32). In addition to increased frequencies of T_regs_, circulating monocytes showed elevated expression of PD-L1 (B7-H1) and tumor-associated macrophages from glioblastoma tissue specimens displayed even greater PD-L1 expression (33). Recent studies now performed in depth flow cytometry studies to characterize the immune exhaustion in the tumor microenvironment. Here, work by Woroniecka et al. and our group showed that exhaustion is a primary feature of the tumor environment and is characterized by the expression of surface markers PD-1, Tim-3, CD39, TIGIT, Lag-3, CTLA-4, 2B4, and BLTA (6, 34). Exhaustion varied according to tumor type, but was especially pronounced in glioblastoma (34). Dysfunctional T cells demonstrated an effector- and transitional memory phenotype indicating antigen-induced activation thereby highlighting that TILs in glioblastoma represent a subpopulation which presumably have been activated to form a tumor-specific response (6).

So far, it is not clear which T cells could benefit from immune checkpoint inhibition, e.g., anti-PD-1 treatment, as currently tested in phase III clinical trials in glioblastoma (35). Although murine studies have pointed out the efficacy of immune checkpoint inhibition using anti-PD-1, anti-Tim-3, anti-CTLA4, combinations thereof and radio-chemotherapy (36, 37), it is incompletely understood which T cell population will be activated and how tumor-specific activation will be constituted (38). In the CD4^+^ TIL compartment, *ex vivo* analyses revealed defined exhaustion profiles of PD-1^+^ cells, which were refractory to PD-1 blockade (39). Taken together, these studies show that T cell dysfunction in the local tumor microenvironment is not yet fully understood, but presumably poses a major obstacle for the formation of a tumor-specific immune response. We hypothesize, that treatment strategies that combine targeted immune activation and T cell disinhibition will most likely be necessary to overcome the challenge of T cell exhaustion.

**Figure 1:**
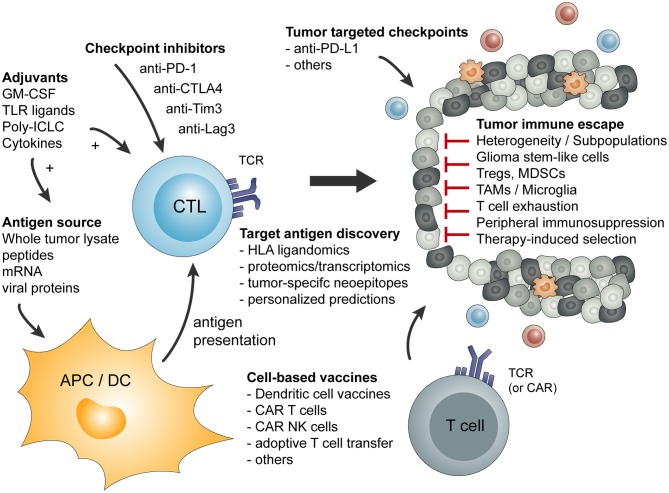
Overview of basic principles of tumor-specific immune activation and the involved cell types. In addition, a short summary of tumor-mediated mechanism of immune escape or immune suppression is given.

**Figure 2:**
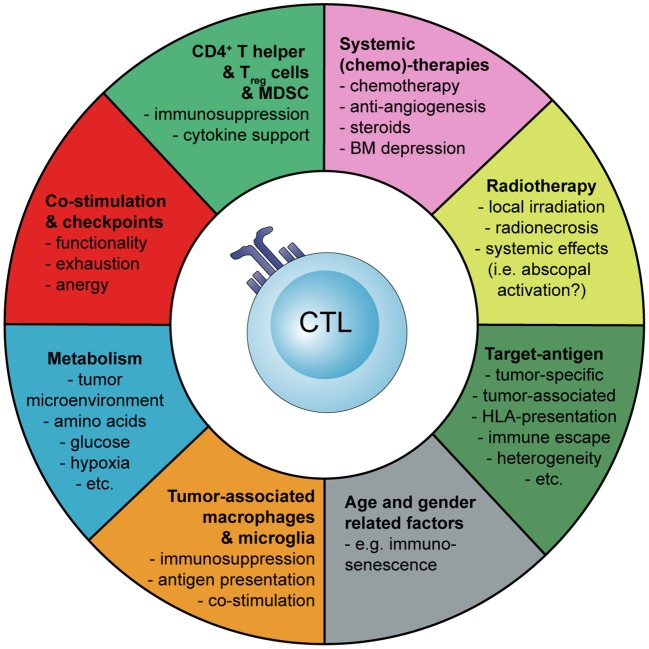
Schematic representation how the activation and tumor-specific response of cytotoxic CD8+ T cells (CTLs) can be influenced during cancer immunotherapy of malignant brain tumors. Myeloid-derived suppressor cells (MDSC), bone marrow (BM).

Basic principles and considerations of tumor-specific immune activation against malignant brain tumors are summarized in Figure 1. Factors influencing tumor-specific cytotoxic CD8^+^ T cells are shown in Figure 2.

## Genetic Landscape in Malignant Brain Tumors

### Mutational Load and Cancer Immunotherapy

Immunotherapy using checkpoint inhibitors has demonstrated remarkable remissions in patients with melanoma and other entities (40–43). However, the long-term therapy response with sustainable anti-tumor responses was limited to a certain subgroup of patients. These patient responses are summarized in the “immunotherapy tail.” Following studies focused on identifying predictive factors for immunological success of tumor-specific response. While the analysis of PD-L1 expression on tumor cells seems not sufficient to predict success of anti-PD-1 checkpoint inhibition (44), recent work in melanoma, colorectal- and lung cancer convincingly identified the mutational load of tumors as significant predictors for response to checkpoint inhibitors (45–47). A higher mutational burden in tumors contributes to increased expression of neo-antigens, which are not expressed in normal tissue, and therefore can be recognized as “foreign,” resulting in tumor-specific immune activation (46). Analysis of matched pretreatment and resistant tumor samples from NSCLC patients during checkpoint inhibition therapy showed that resistant tumor samples displayed a loss of 7 to 18 putative mutation-associated neoantigens in resistant tumors, implicating elimination of specific tumor subpopulations due to T cell activation (48). Unfortunately, the comprehensive computational analysis of mutational events and distribution among multiple cancer entities by Alexandrov et al. revealed that the included brain tumors, i.e., glioblastoma, medulloblastoma, and pilocytic astrocytoma, harbor mutations only at a very low frequency (49). While melanoma, as the entity with the highest mutational load, on average contains >10 mutations per megabasepair (mbp), brain tumors have <1 mutation per mbp (Glioblastoma: 0.9; Medulloblastoma: 0.5 and pilocytic astrocytoma: <0,1 mutations/mbp). As a result, less neo-antigens are available to be recognized by T cells and these tumors are described as immunologically “cold.”

### Mutational Load in Glioblastoma

Selective targeting of essential pathways to inhibit tumor progression has proven ineffective in glioblastoma. Although few core pathways, namely EGFR, RTK/PI3K, p53 and RB regulation, are suspected as initial drivers of proliferation and tumor initiation (50, 51), established glioblastoma diversify into multiple subclonal populations, rendering glioblastoma a highly heterogenic cancer (52). While glioblastoma in the rare childhood cancer syndromes with biallelic mismatch repair deficiency (bMMRd) display a hypermutated phenotype with up to 16-times higher neoantigen load than immunoresponsive melanoma (53), the frequency of neo-antigens in adult newly-diagnosed glioblastoma is low, as high mutational loads are only observed in ~3.5% of tumors (54). Several factors can influence the mutational load of tumors. While smoking-induced lung cancer and UV-associated melanoma are primarily cause by DNA-damaging molecular events, increasing the general mutational burden, no such causes are suspected in glioblastoma pathogenesis. The only factors, known so far, to potentially increase the neoantigen load in glioblastoma are age and the adjuvant treatment consisting of chemo- and radiation therapy. Studies suggest that age-associated mutational burden doubles every 8 years (55). However, in these analyses the general doubling time of different tissue types have to be considered. Therefore, glioblastoma neoantigen load will be only marginally affected, as cells of the central nervous system divide less frequently compared to, e.g., cells of the gastrointestinal tract. Temozolomide, an alkylating agent and first line chemotherapy in newly-diagnosed glioblastoma, promotes the occurrence of mutational events and therefore the frequency of neoantigens amenable for tumor-specific T cell activation. Therapeutic efficacy of temozolomide is closely linked to the presence of promotor methylation of the O6-methylguanine-methyltranferase (MGMT) (56). A recent study be Wang et al. demonstrated that a hypermutation genotype was only found in recurrent glioblastoma and was present in ~17% of cases (57). Interestingly, 94% of tumors with a hypermutated genome gained mutations in genes encoding DNA mismatch repair (MMR) proteins, such as *MSH, PMS*, and *MLH* genes (57). It is unclear if the increased neoantigens load in recurrent glioblastoma is presented to the immune system and can be translated into immunological response to immunotherapy in the recurrent disease stage, as the tumor-specific T cell repertoire appears to loose diversity (6). Interestingly, in contrast to most cancer entities, in which higher mutational burden correlates with better survival, glioma seems to be an exception, as the study by Samstein et al. demonstrated the opposite (58). Does this observation maybe reflect the immuno-oncological privilege of the brain? Currently, the concept of the correlation of a higher mutational burden with improved immunotherapy response is, at least in part, challenged (59). One hypothesis states, that an increased mutational burden might also result in decreased abundance for specific antigens which potentially fall below the threshold of T cell recognition (46, 60). Future studies in brain tumors will have to assess how the mutational burden influences the antigenic landscape and T cell recognition during immunotherapy.

### Genetic Mutations and Heterogeneity in Glioblastoma

The most frequent genomic alterations in glioblastoma are found in *EGFR, PTEN, TP53, TERT*, or *RB1* genes, among others (61). In rare cases and mostly in childhood or adolescent gliomas, additional mutations in histones and chromatin remodeling genes are described (62). However, the molecular pathology of glioblastoma is not defined by point mutations, but rather by copy number variations leading to amplification of genetic drivers in proliferative pathways (61). With the increasing depth of molecular cytogenetics, glioblastoma revealed its complex heterogeneity on a cellular and molecular level. Single cell analysis of *EGFR* amplification, for example, shows that multiple *EGFR* amplifications frequently coexist within the same tumor (51). The presumable molecular subgroup classification into proneural, mesenchymal and classical of individual tumors attempted to define molecular subgroups in order to better understand the driver pathways in glioblastoma (63, 64). However, this molecular profiling was challenged when single cell sequencing demonstrated, that multiple different molecular subtypes can be found within a single tumor (52). Furthermore, the heterogeneous nature of glioblastoma is not only defined by intratumoral, but also intertumoral diversity, as geographically separated, multifocal, or long-term recurrent tumors arise from different clonal subpopulations (65). Overcoming this heterogeneity poses major challenges to tumor-specific immunotherapy strategies, as finding a suitable target to activate T cells against a large diversity of different subclones is complicated (2). Over the next paragraphs we will discuss current efforts to design vaccines which aim to mount a diverse, but tumor specific T cell-mediated immune response against malignant brain tumors.

## Antigenic Landscape in Malignant Brain Tumors and Target Definition

A major challenge of T cell-based immunotherapy is the definition of meaningful targets and several strategic approaches exist to tackle this challenge. It can be hypothesized, that failure of anti-PD-1 trials in glioblastoma so far, is the result of a missing targeted immune response, as immune checkpoints release the breaks on all immune cells with the checkpoint expressed on its surface, regardless of its antigen specificity. Target definition is therefore of utmost importance since the presence of the antigen on tumor cells is a prerequisite for an effective immune-mediated tumor cell lysis. The antigenic structure which is recognized by the TCR is in principle a peptide derived from a source protein presented by an HLA molecule. The following approaches of antigen-discovery are commonly applied.

### Proteomics/Transcriptomics

Definition of candidate antigens are commonly based on precursor data such as proteomic or transcriptomic data. Over- or exclusively expressed genes or proteins are considered potential candidate antigens and peptides with high binding affinity to HLA molecules are predicted using *in silico* algorithms such as SYFPEITHI or netMHC (66, 67). Predicted HLA ligands are usually validated using functional T cell assays in order to demonstrate a memory T cell response in tumor patients. However, this approach has several potential pitfalls including the definition of exclusively or overexpressed genes/proteins. This definition is commonly based on queries using publicly available databases for normal tissue expression levels such as GTEx for gene expression or The Human Protein Atlas for protein levels in various normal human tissues (68, 69). Also, if no primary experimental data of cancer tissue is available, public domain databases such as the The Cancer Genome Atlas (TCGA) are commonly used (70). A conceptual problem in this strategy is the poor correlation of precursor data to direct mass spectrometry-based evidence of the natural HLA ligands. It was shown repeatedly that the natural peptide ligandome presented by HLA does not completely reflect the transcriptome or the proteome (71–74). Another drawback is the uncertainty which is added by using *in silico* epitope prediction algorithms such as SYFPEITHI or netMHC (66, 67). SYFPEITHI is a prediction algorithm which is based on direct mass spectrometry data of eluted peptides from mono-allelic cells and uses position-specific scoring matrices (PSSM) (66, 75, 76).

Other prediction algorithms such as netMHC and netMHCpan are based on machine learning algorithms trained with experimental data from HLA binding assays. A drawback of these training sets is that peptide-MHC complexes displaying weak binding affinities are also included, but they would not necessarily represent physiological interactions. Furthermore, binding assays show that peptides can bind to HLA molecules *in vitro*, but do not take into account any intracellular processing preferences. However, netMHCpan-4.0 integrates both publicly available HLA ligandomics data and binding affinity data, thus increasing the sensitivity and specificity of their binding prediction (67).

Another group of potential immunotherapy targets are mainly defined by a characteristic gene expression profile. So called cancer testis genes encode for a group of immunogenic proteins entitled cancer testis antigens (CTA) which in theory are almost exclusively expressed in the immunoprivileged tissue of the testes, but importantly also in various human malignancies. This unique gene expression pattern led to CTA, such as MAGE, NYESO, IL13Rα2 (77, 78), being considered prime targets for antigen-specific immunotherapy and CTA are targets of ongoing clinical trials in glioblastoma (e.g., NCT02208362; www.clinicaltrials.gov). A publicly available database named CTDatabase (http://www.cta.lncc.br/) lists CTAs. However, it has to be considered that by far not all genes listed fulfill the theoretical criteria of testes- (and tumor-) exclusive expression. In many cases, significant gene expression in normal tissues (i.e., when using the human normal tissue gene expression atlas GTEx) can be detected and the representation of the gene products on HLA of the CTA list members is in general low and not tumor-exclusive (79). Although the CTA concept is interesting from an immunotherapy/antigen discovery standpoint, the definition and listing of CTAs needs major refinement.

Antigens associated with gliomas that derive from “self,” non-mutated sources are best exemplified by the ICT-107 vaccine. ICT-107, is an autologous dendritic cell immunotherapy targeting six “self” antigens with known expression on both tumor and cancer stem cells (80, 81). The antigens in this vaccine are either HLA-A^*^01 restricted (MAGE-1, AIM-2) or HLA-A^*^02 restricted and derive from the following source proteins: melanoma-associated antigen-1 (MAGE-1), antigen isolated from immunoselected melanoma-2 (AIM-2), human epidermal growth factor receptor-2 (HER2/neu), tyrosinase-related protein-2 (TRP-2), glycoprotein 100 (gp100), and interleukin-13 receptor alpha 2 (IL-13Rα2). The selection is based on preclinical studies including gene expression analysis, direct evidence of the source protein as well as the detection of T cell responses against the mentioned antigens (82–89). Regarding inter-tumoral heterogeneity, at least 3 antigens were expressed on all of the tumors, four or more antigens on 97% of tumors, and all 6 antigens were expressed in 83% of tumors. The exact intra-tumoral heterogeneity of these antigens within the tumor is not known, however targeting multiple epitopes rather than a single epitope should decrease the probability of tumor escape. In a phase I study, ICT-107 was well-tolerated and could induce systemic type I cytokine responses (80). A phase II study did not meet the primary endpoint of improved overall survival, however it showed significantly improved progression-free survival with maintained quality of life and most interestingly a clinical and immunological benefit for patients that were HLA-A^*^02 positive (90). Unfortunately, an already started phase III trial was suspended due to financial problems of the industry sponsor.

### Neoepitopes

Neoepitopes, as described above, are derived from somatic cancer mutations and thus tumor-specific. They contain “foreign” sequences and are believed to be highly immunogenic as they were reported to contribute to relevant immune responses (91–97). Due to this relative immunogenicity, memory T cell responses based on *in silico* neoepitope predictions could just be a relic of immunoediting (27). If the established tumor was shaped by immune selection of antigen-loss variants, then *in silico* predictions, even if supported by memory T cell data, fall short to overcome this immune escape mechanism. Nevertheless, it has to be taken into consideration that two phase I clinical trials use *in silico* neoepitope predictions in melanoma patients induced immune responses. Mutated epitopes were targeted by fully individualized RNA (NCT02035956) or long peptide vaccines (NCT01970358) based on up to 20 *in silico* predicted neoepitopes (96, 97). Both approaches induced or boosted neoantigen-specific T cell responses also associated with a clinical response (96, 97). This further emphasizes the potential of neoantigens as targets for peptide-specific immunotherapies (96, 97). However, in contrast to melanoma, the mutational load in malignant brain tumors is rather low and the likelihood of such an approach to lead to clinical success in brain tumors is limited. Thus, in malignancies with low mutational load such as glioblastoma, it seems to be important to apply antigen discovery strategies that also incorporate non-mutated targets.

### HLA Ligandome Mapping Using Mass Spectrometry

An alternative strategy to bottom-up strategies that start from genomic, transcriptomic or proteomic data is the direct analysis of the peptides that are naturally presented by HLA. The most commonly used technique is immunopurification of peptides followed by mass spectrometry (LC-MS/MS) sequencing. As opposed to the epitope prediction algorithms mentioned above, mapping the natural HLA ligandome is an unbiased technique and describes the immunologically pivotal level of peptide presentation without the need of inferring information from precursor data. This strategy was pioneered and systematically developed by a research group at the Department of Immunology at the University of Tübingen in Germany, led by Hans-Georg Rammensee and Stefan Stevanović. Early efforts were still limited by much lower technical sensitivity of the mass spectrometry devices available and the need for manual fragment spectra interpretation, also requiring larger amounts of tissue samples. Technical improvements of mass spectrometers in mass accuracy, sensitivity, resolution and speed as well as the miniaturization of the immunopurification protocols led to the generation of large datasets and the possibility to map the HLA ligandome of smaller amounts of tissue. At the same time, bioinformatic tools were developed to process the growing amount of mass spectrometry data, introducing automatic fragment spectrum annotation and to deconvolute multi-allelic datasets (98). Target definition for cancer immunotherapy using this approach is usually based on comparative profiling of tumor against benign HLA ligandome data. Potential non-mutated targets are characterized by frequent representation in malignant, but not in benign immunopeptidomes (99–102). Tumor-exclusivity can either be on the level of HLA ligands, e.g., in terms of differential antigen processing in cancer cells (103), or on the level of the entire antigen (79, 99, 101, 102, 104). So far, in-depth investigations of the non-mutated immunopeptidome presented on native clinical samples have been published for AML (99), CLL (105), MM (102), mantle cell lymphoma (93), EOC (101), and metastatic malignant melanoma. Regarding glioblastoma, the first HLA peptidome publications described the HLA-ligandome of HLA-A^*^02 (the most frequent HLA class I allotype in Caucasians) including immunogenicity testing of the most promising candidates and the ligandome of non-HLA-A^*^02 allotypes (106, 107). Both authors used gene expression data as an adjunct for target selection despite the known uncoupling of transcriptome/proteome and the HLA ligandome. The HLA class II ligandome of patient samples and was first described in a paper that focused on the description of the antigenic landscape of glioblastoma stem-like cells (79). In this publication, which also included immunogenicity testing of candidates, antigen definition was based on a purely HLA ligandome centric approach using a comparative analysis of tumor samples and a large database of HLA peptidomes of various normal tissues. An HLA peptidome paper that used 3 glioblastoma cell lines could show the induction of CTAs by the chemotherapeutic drug decitabine, which however is not commonly used to treat glioblastoma (71). A recent analysis of plasma-soluble HLA ligands found a strong correlation between plasma ligands and tumor tissue derived ligands, but a weak correlation between both plasma and tumor tissue derived peptidomes and the tumors proteoms (108).

### The Importance of Choosing the Appropriate Tissue Sample

An important question arises even before choosing one of the antigen-discovery strategies mentioned above. Irrespective of the technique, results will always depend on the tissue input. Glioblastoma is a highly heterogeneous tumor and different tumor regions such as a mainly necrotic core, a contrast-enhancing, highly vascularized rim, and a non-enhancing infiltration zone at the peripheral border of the lesion (61). Even within these regions, intratumoral heterogeneity calls for a multi-epitope approach since various antigens are only present in certain subclones. This is especially true for common glioblastoma mutations such as the mutations of the epidermal growth factor receptor (EGFR). The EGFR gene is amplified in roughly 40% of glioblastomas and patients with EGFR amplified tumors frequently have a deletion mutation called EGFRvIII (109). The EGFRvIII mutation occurs in 20–30% of all glioblastoma patients, but even if the mutation is present, only a fraction of tumor cells will be positive (110). This example highlights the importance of tissue sampling, ideally with biological replicates and with samples from various tumor regions to prevent sampling errors and to enlarge the variety of tumor antigens that can potentially be discovered. Also, analytic techniques should reflect tumor heterogeneity (e.g., single cell sequencing or next generation sequencing techniques with high sequencing depth/coverage).

Another aspect that has to be considered is the tumor cell content in brain tumor samples. A surgical biopsy will always be “contaminated” by non-tumorous cells such as infiltrating immune cells, endothelial cells, normal neuronal or glial tissue or peripheral blood cells. Several strategies exist to address this issue. For cleaner sampling tissue digestion of the surgical sample can be performed in order to receive a single cell suspension, which then will be sorted to exclude certain fractions (e.g., CD45^+^ immune cells or CD31^+^ endothelial cells) before further analysis. For example, fractionated HLA class I immunoprecipitation of cell populations from enzymatically dissociated ovarian cancer was performed with high peptide yields (101). However, a major problem of malignant brain tumors is the lack of a robust surface marker that can be used for positive selection of glioblastoma cells. Thus, tumor cells can only be enriched by depleting other cell fractions. Another strategy to correct for non-tumorous cells in patient samples are comparative analyses with long-term cell lines. Long-term cell lines will consist of 100% tumor cells, but are prone to cell culture artifacts over time. However, comparative analyses with patient samples and focusing on antigens that are both present in the long-term cell line and in the fresh surgical specimen can lower the likelihood of detecting antigens from non-tumorous cells or antigens that are due to the artificial environment of cell culture (79). Despite the attempt to deconvolute tumor heterogeneity on a non-hierarchical level, it has to be considered that certain cell populations within the tumor bulk might represent high priority targets. In a hierarchical tumor like glioblastoma, a small subpopulation of tumor cells might display a more malignant phenotype and exhibit stem cell-like functions. These cells are termed glioma stem-like cells (GSC) and play a crucial role in tumorigenesis, tumor maintenance and resistance toward conventional therapies such as radiotherapy and chemotherapy (61). Therefore, the antigenic landscape of this subpopulation is of great immunotherapeutical interest. Recently, GSC antigens have been described based on proteomics and based on HLA ligandomics (79, 107, 111).

## Vaccine Studies in Glioblastoma

**Figure 3:**
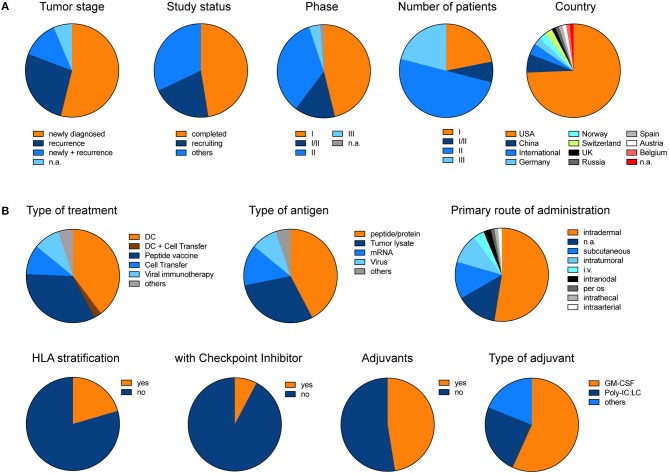
Pie charts illustrate the different trials listed under www.clinicaltrials.gov when searching for “glioblastoma” AND “vaccine.” Terminated, trials with unknown status or withdrawn trials were excluded. **(A)** General and **(B)** detailed overview of different clinical trials. Charts illustrate the data which is summarized in Supplementary Table 1. The data was last updated 11/2018.

In order to give an overview of currently ongoing and already recruited vaccine trials in glioblastoma, we summarized all trials from www.clinicaltrials.gov (Supplementary Table 1). Excluding trials with a “terminated, “unknown” or “withdrawn” status, we found 78 trials under the search term “glioblastoma” and “vaccine” which in total aim to recruit 4130 patients (Figure 3). Overall 54% of trials primarily investigate newly diagnosed glioblastoma. Although only 4% of trials so far reached the phase III, these trials will include 21% of all patients (Figure 3A). The two most prominent treatment approaches are dendritic cell vaccines and peptide vaccinations. The primary rout of vaccination was intradermally. Intratumoral injection or local therapy only represented a fraction of all primary treatment routes. Interestingly, so far, only very few trials incorporated HLA stratification of patients or combined their vaccination strategy with checkpoint inhibitors, and only about 50% of trials applied adjuvants (Figure 3B).

In addition, currently there are 39 phase I studies found under the term “glioblastoma” and “immunotherapy” at www.clinicaltrials.gov which are recruiting, not yet recruiting, enrolling by invitation, or active (not recruiting). These trials are increasingly integrating combinatorial treatment approaches which apply a vaccination or cellular-based, i.e., CAR T cells, approach together with a checkpoint inhibitor in order to increase the efficacy of the tumor-specific T cell response (Table 1). Genetically modified T cells are currently being evaluated in multiple trials. For example, CAR T cells specific for the IL13Rα2 are genetically optimized for improved co-stimulation during activation (Table 1). Better understanding of the multifaceted biology of tumor-specific immune responses will allow, for example, gene therapeutic techniques to be increasingly utilized to specifically modulate and improve tumor vaccines.

**Table 1:** Overview of the current phase I study landscape for the search term “glioblastoma” AND “immunotherapy” (last updated 01/2020, *n* = 39 studies, www.clinicaltrials.gov).

**NCT identifier**	**Status (01/2020)**	**Phase**	**Estimated enrollment**	**Tumor type**	**Study title**
NCT02649582	Recruiting	I/II	20	Newly diagnosed GBM	Adjuvant dendritic cell-immunotherapy plus temozolomide in GBM patients (ADDIT-GLIO)
NCT04165941	Not yet recruiting	I	12	Newly diagnosed GBM	Novel gamma-delta (γδ)T cell therapy for treatment of patients with newly diagnosed GBM (DRI)
NCT03961971	Not yet recruiting	I	15	Recurrent GBM	Trial of anti-tim-3 in combination with anti-PD-1 and SRS in recurrent GBM
NCT03426891	Recruiting	I	32	Newly diagnosed GBM	Pembrolizumab and vorinostat combined with temozolomide for newly diagnosed GBM
NCT00639639	Active, not recruiting	I	42	Newly diagnosed GBM	Vaccine therapy in treating patients with newly diagnosed GBM multiforme
NCT03707457	Recruiting	I	30	Recurrent GBM	Biomarker-driven therapy using immune activators with nivolumab in patients with first recurrence of GBM
NCT02208362	Recruiting	I	92	Recurrent °III or °IV glioma	Genetically modified T-cells in treating patients with recurrent or refractory malignant glioma
NCT04047706	Recruiting	I	30	Newly diagnosed GBM	Nivolumab, BMS-986205, and radiation therapy with or without temozolomide in treating patients with newly diagnosed GBM
NCT04201873	Not yet recruiting	I	40	Recurrent GBM	Pembrolizumab and a Vaccine (ATL-DC) for the treatment of surgically accessible recurrent GBM
NCT04003649	Recruiting	I	60	Recurrent GBM	IL13Ralpha2-targeted chimeric antigen receptor (CAR) T cells with or without nivolumab and ipilimumab in treating patients with recurrent or refractory GBM
NCT03714334	Active, not recruiting	I	24	Recurrent GBM	DNX-2440 oncolytic adenovirus for recurrent GBM
NCT03170141	Enrolling by invitation	I	20	Recurrent GBM	Immunogene-modified T (IgT) cells against GBM
NCT02852655	Active, not recruiting	I	35	Recurrent GBM	A pilot surgical trial to evaluate early immunological pharmacodynamic parameters for the PD-1 checkpoint inhibitor, pembrolizumab (MK-3475), in patients with surgically accessible recurrent/progressive GBM
NCT03491683	Active, not recruiting	I / II	52	Newly diagnosed GBM	INO-5401 and INO-9012 Delivered by electroporation (EP) in combination With cemiplimab (REGN2810) in newly-diagnosed GBM
NCT03174197	Recruiting	I / II	60	Newly diagnosed GBM	Atezolizumab in combination with temozolomide and radiation therapy in treating patients with newly diagnosed GBM
NCT03389230	Recruiting	I	42	Recurrent GBM	Memory-enriched t cells in treating patients with recurrent or refractory grade III-IV glioma
NCT03344250	Recruiting	I	18	Newly diagnosed GBM	Phase I EGFR BATs in newly diagnosed GBM
NCT03347097	Recruiting	I (early)	40	Newly diagnosed GBM	Adoptive cell therapy of autologous TIL and PD1-TIL cells for patients with GBM
NCT03158389	Recruiting	I / II	350	Newly diagnosed GBM	NCT Neuro master match - N^2^M^2^ (NOA-20) (N^2^M^2^)
NCT03866109	Recruiting	I / II	21	Newly diagnosed GBM	A phase I/IIa study evaluating temferon in patients With GBM & unmethylated MGMT (TEM-GBM)
NCT03392545	Recruiting	I	30	Malignant glioma	Combination of Immunization and Radiotherapy for Malignant Gliomas (InSituVac1) (InSituVac1)
NCT03341806	Recruiting	I	30	Recurrent GBM	Avelumab With laser interstitial therapy for recurrent GBM
NCT03532295	Not yet recruiting	I / II	55	Recurrent GBM	Epacadostat in combination with radiation therapy and avelumab in patients with recurrent gliomas
NCT02529072	Active, not recruiting	I	7	Recurrent °III or °IV glioma	Nivolumab with DC vaccines for recurrent brain tumors (AVERT)
NCT02062827	Recruiting	I	36	Recurrent °III or °IV astrocytoma	Genetically engineered HSV-1 phase 1 study for the treatment of recurrent malignant glioma (M032-HSV-1)
NCT03223103	Recruiting	I	20	Newly diagnosed GBM	Safety and immunogenicity of personalized genomic vaccine and tumor treating fields (TTFields) to treat GBM
NCT02766699	Recruiting	I	20	Recurrent GBM	A study to evaluate the safety, tolerability and immunogenicity of EGFR(V)-EDV-Dox in subjects With recurrent GBM (CerebralEDV)
NCT02010606	Active, not recruiting	I	39	Newly diagnose & recurrence GBM	Phase I study of a dendritic cell vaccine for patients with either newly diagnosed or recurrent GBM
NCT02502708	Recruiting	I	115	Malignant brain tumor	Study of the IDO pathway inhibitor, indoximod, and temozolomide for pediatric patients with progressive primary malignant brain tumors
NCT03619239	Recruiting	I / II	18	Newly diagnosed GBM	Dose-escalation study to evaluate the safety and tolerability of GX-I7 in patients with GBM
NCT03382977	Recruiting	I / II	38	Recurrent GBM	Study to evaluate safety, tolerability, and optimal dose of candidate GBM vaccine VBI-1901 in recurrent GBM subjects
NCT03657576	Recruiting	I	24	Recurrent GBM	Trial of C134 in patients with recurrent GBM (C134-HSV-1)
NCT03043391	Recruiting	I	12	Malignant glioma (children)	Phase 1b Study PVSRIPO for recurrent malignant glioma in children
NCT03576612	Recruiting	I	36	Newly diagnosed HGG	GMCI, nivolumab, and radiation therapy in treating patients with newly diagnosed high-grade gliomas (GMCI)
NCT03152318	Recruiting	I	108	Recurrent malignant glioma	A Study of the treatment of recurrent malignant glioma with rQNestin34.5v.2 (rQNestin)
NCT03911388	Recruiting	I	15	Recurrent cerebellar brain tumors	HSV G207 in children with recurrent or refractory cerebellar brain tumors
NCT03058289	Recruiting	I / II	110	Multiple cancers	A phase 1/2 safety study of intratumorally dosed INT230-6 (IT-01)
NCT02457845	Recruiting	I	18	Recurrent supratentorial brain tumor	HSV G207 alone or with a single radiation dose in children with progressive or recurrent supratentorial brain tumors
NCT00634231	Active, not recruiting	I	12	Malignant glioma or recurrent ependymoma	A phase I study of AdV-tk + prodrug therapy in combination with radiation therapy for pediatric brain tumors

*Only trials with the following status are listed: early phase I, phase I, recruiting, not yet recruiting, active not recruiting and enrolling by invitation. Please refer to clinicaltrials.gov and the official identifier for detailed information on each clinical trial*.

## Clinical Application in Personalized Cancer Immunotherapies and Design of Future Trials

High degrees of inter- and even intra-patient heterogeneity in tumor biology demand tailored therapies. Personalized cancer immunotherapy can be divided into three categories. Stratified approaches include biomarker-based selection of patients subsequently treated with the same drug (e.g., rindopepimut) in the presence of the EGFRvIII mutation. Passively personalized therapies are based on autologous cellular material, whereby tumors did not undergo molecular characterization (e.g., whole tumor lysates). In turn, actively personalized approaches do not only apply molecular markers for patient selection, but also for definition of drug composition. Active personalization can be categorized into warehouse and fully individualized concepts, explained at the example of peptide vaccination. Warehousing includes selecting off-the-shelf peptides for individual vaccine cocktails, whereas fully individualized therapies depend on *de novo* synthesis of patient-specific peptides identified by immunopeptidomics, epitope prediction or immunogenicity screening (112).

Regarding personalized immunotherapy approaches targeting glioblastoma, two early phase studies were published recently which are great examples of current strategies in this field. A phase I/Ib study applied a neoantigen multiepitope peptide vaccine for patients with newly diagnosed MGMT-unmethylated glioblastoma (113). Resected tumor tissue DNA and normal germline DNA were analyzed to identify neoantigens and vaccine production occurred during recovery from surgery and administration of radiotherapy. Vaccines contained up to 20 long peptides that were administered in a prime–boost schedule with poly-ICLC (polyinosinic and polycytidylic acid) following radiotherapy. A total of 8 patients were vaccinated and in 5 patients, tissue from a recurrent tumor surgery could be analyzed. Although *in silico* prediction was based on an HLA class I algorithm, both neoepitope-specific CD8^+^ and CD4^+^ T cell responses were found in the periphery and among tumor-infiltrating lymphocytes. Interestingly, immune responses were only observed in patients that did not require steroid treatment. As many brain tumor patients receive potent steroids such as dexamethasone to treat perilesional edema, immunotherapists have to acknowledge the known immune suppression linked to glucocorticoids and come up with alternative strategies to manage brain edema. Such strategies might include the use of bevacizumab.

In a phase I trial published by the Glioma Actively Personalized Vaccine Consortium (GAPVAC) 15 patients with newly diagnosed glioblastoma (HLA-A^*^02:01- or HLA-A^*^24:02-positive) were treated with a peptide vaccine (APVAC1) derived from a premanufactured warehouse of unmutated antigens followed by APVAC2 mainly targeting *in silico* predicted neoepitopes. Personalization was based on mutations and analyses of the transcriptomes and HLA ligandome of patient tumors. Immunogenicity for both APVAC1 and APVAC2 was shown. Unmutated APVAC1 antigens triggered predominantly central memory CD8^+^ T-cells responses, while APVAC2 induced mainly CD4^+^ T cell responses.

Both studies showed feasibility and safety—vaccines based on personalization and rather challenging logistics could be produced and administered in a meaningful time. Both studies also showed antigen-specific immunogenicity, however it remains still unclear whether these strategies will lead to improved patient survival in the future.

The vaccination trial by Keskin et al. found expression of multiple co-inhibitory receptors on post-vaccination TIL—consistent with T cell exhaustion (113). These findings warrant the investigation of combining the strategies mentioned above with immune checkpoint blockade.

We hypothesize, that for successful future clinical trials two major immunological concepts primarily need to be taken into consideration: (1) Guiding the immune system toward mounting a tumor-specific immune response, i.e., defining the tumor as the target, (2) Boosting the tumor-specific immune response to be able to overcome tumor-mediated immunosuppression and immune escape. For point 1, multiple therapeutic treatment options, including peptide vaccines, tumor lysates, RNA vaccines, DC vaccines, oncolytic viruses, autologous cell transfer, CAR T cells, NK cells and others, will provide the basis to elicit an immune response which specifically targets tumor cells. However, these treatment approaches will need to be combined with additional adjuvants that are able to support the formation and execution of a tumor-specific immune response (point 2) in order to overcome the immunosuppressive microenvironment, metabolic challenges, immune dysfunction, and exhaustion as well as peripheral immunosuppression. Such adjuvants can be ceckpoint inhibitors, inhibitors of local immunosuppressive pathways and other stimulants of the immune system such as cytokines or TLR ligands. Molecular profiling of the tumor microenvironment, expressional- and mutational subgroups, as well as urgently needed studies on the interaction of tumor-specific immune treatment approaches with the current standard therapy regiments, i.e., radio- and chemotherapy, will further improve the knowledge of the complex immune dynamics in malignant brain tumors and subsequently the efficacy of upcoming tumor-specific immunotherapy.

## Conclusion

Taken together, our review highlights the current obstacles to determine the optimal path to therapeutically mount a tumor-specific immune response for patients with malignant brain tumors. Recent advances in antigen discovery and personalization will be supported by the increasing use of checkpoint inhibition, potentially leading to a breakthrough of tumor vaccines.

## Author Contributions

MM and MN both contributed equally to this review.

### Conflict of Interest

The authors declare that the research was conducted in the absence of any commercial or financial relationships that could be construed as a potential conflict of interest.

## Supplementary Material

Supplementary Table 1 The table summarizes the search results of “(glioblastoma) and (vaccine)” on clinicaltrials.gov, last updated 11/2018. We excluded terminated trials, trials with unknown status, withdrawn studies and non-vaccine or immunostimulatory trials. We do not claim that this table displays a complete overview of all clinical trials for this topic and therefore sincerely apologize if additional trials were missed.

## Abbreviations

bMMRd: biallelic mismatch repair deficiency
CD: cluster of differentiation
CTA: cancer testis antigen
CTLs: cytotoxic T lymphocytes
EGFR: epidermal growth factor receptor
EGFRvIII: epidermal growth factor receptor variant III
GSC: glioma stem-like cells
HCMV: human cytomegalovirus
HLA: human leukocyte antigen
IDO: indolamine desoxygenase
MGMT: O6-methylguanine-DNA-methyltransferase
NSCLC: non-small cell lung cancer
PD-L1: programmed death ligand 1
TAA: tumor-associated antigen
TCGA: The Cancer Genome Atlas
TCR: T cell receptor
TGFβ: transforming growth factor β
TIL: tumor-infiltrating lymphocytes
Tr1: type 1 regulatory T cells
T_regs_: regulatory T cells
TSA: tumor-specific antigen
WHO: World Health Organization.
